# On Dyspepsia

**Published:** 1877-07

**Authors:** 


### ARTICLE X.

### *On Dyspepsia.*

At a late meeting of the Harveian Society of London, Dr. Farquharson read a paper on this subject.

Attention was directed to the state of the tongue in dyspepsia. A deeply fissured tongue often meant little; whereas a thin white fur, composed of minute dots, was generally found along with pain immediately after food. Pain after a longer interval was accompanied by a pale, flabby tongue, with reddish tip and centre. The treatment of dyspepsia consisted of two parts, that of food and that of drugs. The latter was the principal part with patients applying for gratuitous relief. The pain occurring immediately after food was usually relieved by alkalies; whereas acids were indicated where suffering was not experienced until an hour or two after the commencement of the digestive act. For the relief of the nausea and sickness remaining after the bowels were thoroughly cleansed, nothing was so effectual as hourly drop-doses of ipecacuanha wine. Nux vomica was also a valuable remedy. Pain might be but the protest of the stomach against an overload or be the result of a deficient tone, from general nervous exhaustion. In some cases each meal was followed by diarrhœa; and for these cases attention was directed to Ringer's plan of minute doses of the liquor hydrargyri perchloridi. In speaking of diet, Dr. Farquharson pointed out that there are three forms of dyspepsia: 1. The dyspepsia of fluids, as it is called, where the stomach seems intolerant of all forms of fluid. 2. The digestive derangements following intemperance in the matter of animal food; and, 3. The dyspepsia connected with indulgence in tea, or other warm and weak infusion of tannin.—*Med. and Surg. Rep.*

---

